# Postoperative Neurocognitive Disorders in Cardiac Surgery: Investigating the Role of Intraoperative Hypotension. A Systematic Review

**DOI:** 10.3390/ijerph18020786

**Published:** 2021-01-18

**Authors:** Marcelina Czok, Michał P. Pluta, Zbigniew Putowski, Łukasz J. Krzych

**Affiliations:** 1 Students’ Scientific Society, Department of Anaesthesiology and Intensive Care, Faculty of Medical Sciences in Katowice, Medical University of Silesia, 40752 Katowice, Poland; mczok@poczta.fm (M.C.); putowski.zbigniew@gmail.com (Z.P.); 2 Department of Anaesthesiology and Intensive Care, Faculty of Medical Sciences in Katowice, Medical University of Silesia, 40752 Katowice, Poland; michal_p2@tlen.pl

**Keywords:** intraoperative hypotension, cardiac surgery, postoperative delirium, postoperative cognitive decline, cognitive deficit

## Abstract

Perioperative neurocognitive disorders remain a challenging obstacle in patients after cardiac surgery, as they significantly contribute to postoperative morbidity and mortality. Identifying the modifiable risk factors and mechanisms for postoperative cognitive decline (POCD) and delirium (POD) would be an important step forward in preventing such adverse events and thus improving patients’ outcome. Intraoperative hypotension is frequently discussed as a potential risk factor for neurocognitive decline, due to its significant impact on blood flow and tissue perfusion, however the studies exploring its association with POCD and POD are very heterogeneous and present divergent results. This review demonstrates 13 studies found after structured systematic search strategy and discusses the possible relationship between intraoperative hypotension and postoperative neuropsychiatric dysfunction.

## 1. Introduction

Perioperative neurocognitive disorders include a variety of neuropsychiatric complications, amongst which postoperative delirium (POD) and postoperative neurocognitive disorder (deficit) (POCD) are the most common and clinically important [1]. According to the DSM-V criteria, POD is considered when disturbance of consciousness with reduced ability to focus, sustain and shift attention, and awareness, occurs. It has an acute onset and fluctuating course, usually is self-limiting and resolves within a few days after surgery. Disturbance in cognition, with memory deficit, disorientation, language, visuospatial ability or perception, appears [2]. Diagnosis should be based on complex psychiatric assessment but there are several validated bedside tools with high diagnostic accuracy which can be successfully applied by other clinicians [3,4,5]. The diagnostics of POCD is more challenging and time-consuming because there are no universal international recommendations when and how to diagnose cognitive decline in the postoperative period [6]. POCD affects global cognitive functions for several days, months or even years after surgery and its recognition requires detailed neuropsychological testing which can be based on clinical assessment by psychologists or with a battery of various tests [7]. Precise preoperative assessment is a must to establish reliable diagnosis as neurocognitive disorder makes the evaluation difficult. Also, POD may aggravate the risk of subsequent POCD.

According to recent recommendations, the term ‘perioperative neurocognitive disorders’ encompasses cognitive impairment identified in the preoperative or postoperative period. This includes cognitive decline diagnosed before operation (described as neurocognitive disorder); any form of acute event (postoperative delirium, POD) and cognitive decline diagnosed up to 30 days post-surgery (delayed neurocognitive recovery) and up to 12 months (postoperative neurocognitive disorder, POCD) [1].

Intraoperative hypotension (IOH) has deleterious impact on blood flow and tissue perfusion [8]. The brain and the kidneys are the most susceptible organs for variations of blood pressure (BP) in cardiac surgery procedures. Appropriate or “safe” blood pressure (BP) values before, during and after cardiopulmonary bypass (CPB) are unknown: the mean arterial pressure (MAP) target of 70–100 mmHg is the wide range [9]. The optimal cerebral perfusion pressure based on the cerebral autoregulation, a mechanism that maintains a stable cerebral blood flow in the face of a fluctuating BP both during on-pump and off-pump CABG (coronary artery bypass grafting) surgeries is under debate [10]. It is easy to underestimate or overestimate the lower limit of cerebral autoregulation in an individual patient if it is not directly monitored [10]. Most data regarding the neurological consequences of IOH relate to postoperative stroke [11,12,13]. Stroke is frequently but not always associated with POD or POCD [14]. Unfortunately, there is paucity of high quality evidence and summary data regarding IOH and POD or POCD in general, despite their clear pathophysiological association [15]. 

Recently, Feng et al. published the first meta-analysis regarding IOH and postoperative cognitive impairment [16]. The authors analyzed five randomized controlled trials, in which participants undergoing both cardiac (3 studies) or non-cardiac (2 studies) surgery were divided into low-target and high-target MAP groups. They found no differences in the incidence of POD and POCD between the groups. Although the paper is an important step in exploring the association between IOH and postoperative cognitive impairment, its certain drawbacks such as a very limited number of patients and inconsistent study populations do not allow for definite clarification of the issue.

Based on the abovementioned considerations, we performed a systematic review of the literature evaluating the association between IOH and postoperative neurocognitive disorders in cardiac surgery.

## 2. Materials and Methods 

**Figure 1:**
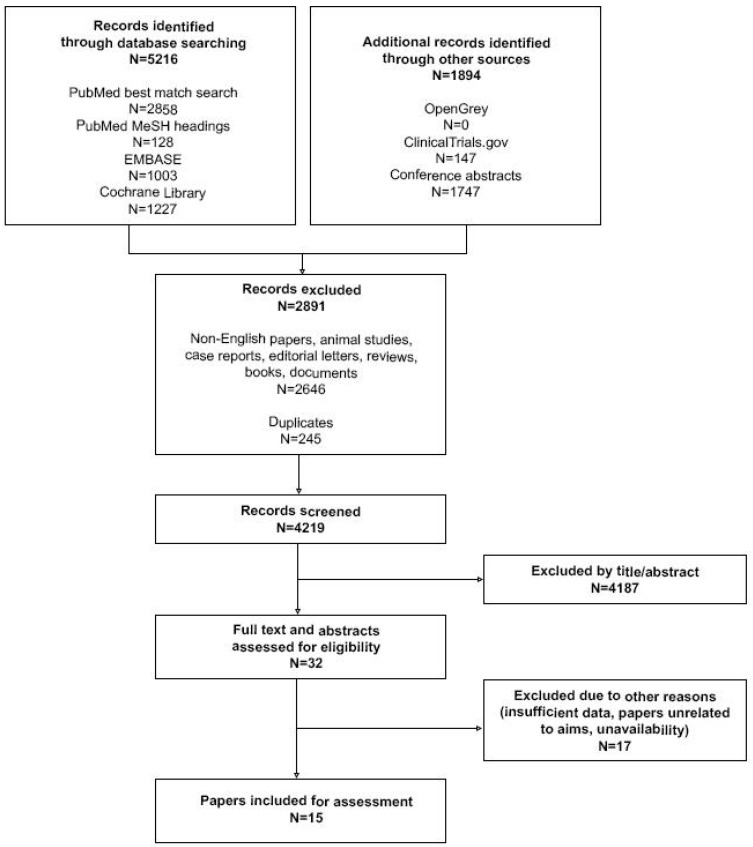
Articles selection process.

To avoid publication bias, we used a structured systematic search strategy of all relevant publications in the field (Appendix A). PRISMA guidelines were used for appropriate reporting. We screened for data that were published till the 6th of January 2021. PubMed, EMBASE and Cochrane Library were screened. Additional search was performed to retrieve grey literature. Then, we excluded animal studies, papers other than in English language and non-original papers. Duplicates were identified and excluded as well. All the remaining abstracts (*n =* 4219) were screened by 4 independent investigators and full texts were retrieved if at least 3 adjudicators agreed to include the paper. Differences of opinion were resolved by discussion. Then, available manuscripts (*n =* 32) were reviewed by two independent investigators And included into comprehensive assessment if two adjudicators agreed that the study results were compliant with the goals of this review (Figure 1). If no agreement was reached, then a third reviewer made a final decision. Due to clear heterogeneity between publications and subsequent risk of bias we failed to prepare meta-analysis.

## 3. Review of Published Data

### 3.1. Postoperative Cognitive Decline

One of the first published investigations in the field was performed by Gold and colleagues [17]. In their study, 248 patients undergoing elective CABG with CPB were randomized into two groups. In the experimental group MAP was maintained during CPB between 80–100 mmHg, while in the control group it was kept within 50–60 mmHg. Vasoactive drugs were administered to keep MAP in the target ranges. Cognitive function was assessed preoperatively, then 7 days and 6 months post procedure using a battery of 11 neuropsychological tests. POCD was defined as a decline in three or more cognitive tests in comparison with preoperative results and was determined a priori by a panel of experts. Even though at 6 months follow-up the high MAP group had significantly lower combined incidence of cardiac and neurologic morbidity and mortality, there were no statistically significant differences in cognitive function between the groups. However, such outcome can be related with very strict criteria for diagnosing cognitive impairment applied by the authors, as in most research a decline in only one or more tests in neurocognitive assessment is considered significant. Moreover, the authors shared only the results at 6 months follow-up, while POCD can occur within the first weeks after the surgery [18].

In the next study, performed as a continuation of the abovementioned one, Charlson et al. aimed to compare two blood pressure management strategies in patients undergoing elective CABG with CPB [19]. 412 patients were randomly assigned to either “custom” MAP group, in which MAP target was determined by patient’s usual pre-bypass blood pressure, or to “high” MAP group, in which MAP was maintained around 80 mmHg. All participants were evaluated before the surgery, on the 1–2 and 5–6 postoperative days and at 6 months follow-up using the same neuropsychological battery as in the prior study. There were no statistically significant differences in cognitive status between subjects, nevertheless the same drawbacks as previously should be taken into consideration in data interpretation. Furthermore, even though the target MAP in the “custom” group varied from 57 to 90 mm Hg, the achieved average MAP corrected for low flow was very similar in both groups (79 mmHg in “custom” vs. 76 mmHg in “high” MAP group), what could have contributed to the lack of significant differences in outcomes.

The next study was a follow-up of randomized controlled Perfusion Pressure Cerebral Infarcts (PPCI) trial in which patients were allocated to either high target (MAP = 70–80 mmHg) or low target (MAP = 40–50 mmHg) blood pressure during CPB [20]. One of the goals of the study was to detect any differences in cognitive function and instrumental activities of daily living between the groups at a 3-year follow-up. Among the 197 patients participating in the primary study 55 in the high target group and 58 in the low target group were eligible for further analysis and examined with MMSE, the International Study of Postoperative Cognitive Dysfunction (ISPOCD) test battery and three additional questionnaires. At a 3-years follow-up POCD occurred in 18.9% and 14.0% in the high-target and low-target groups, respectively; OR = 1.01 (CI 95% 0.33–3.12, *p* = 0.97; adjusted for dropout and age). There were no statistically significant differences between the groups in the subjective level of cognitive function or instrumental activities of daily living as well. Nevertheless, it is worth emphasising that even though dropout adjustment was applied, the proportion of POCD was probably underestimated, as a considerable number of patients (28 at low target and 21 in high target group) did not respond to contact attempts, declined cognitive testing or cancelled the appointments. If POCD was in fact present among those patients with predominance in one of the groups, results could have been adequately different.

Newman et al. conducted a study aimed to establish the association between MAP during CPB, rewarming rate, and cognitive outcome in cardiac surgery [21]. The authors analysed data from 237 patients examined with neuropsychological test battery on the day prior to the surgery and the day prior to the hospital discharge (approximately 7–10 days after surgery). During the surgery MAP was registered automatically at 1 min intervals in order to determine MAP area less than 50 mm Hg (taking time and depth of hypotension into account). Multivariable linear regression revealed that there was no association between intraoperative MAP and cognitive decline. On the other hand, interaction of age with MAP area less than 50 mm Hg was significantly related with cognitive decline in Digit Symbol Test (*p* = 0.005). Even though this study shows that low intraoperative MAP is not a crucial risk factor for POCD, it contributes to neuropsychological dysfunction in elderly. This finding is particularly important, as the population of senile patients undergoing cardiac surgeries is growing [22].

In a small cohort study Gottesman and colleagues investigated the relationship between the change in BP during on-pump CABG and early cognitive dysfunction in 15 patients with high risk for postoperative stroke [23]. Patients were assessed preoperatively, 3–5 days after the surgery and (eleven of them) after 1 month. The neurocognitive examination consisted of MMSE, Trail Making Test A and B and the modified Rankin Scale. Unadjusted analysis of continuous change in MAP demonstrated that each additional point decrease in baseline MAP during the surgery led to a 0.09-point greater decline in early postoperative MMSE score (*p* = 0.02). When probability of stroke calculated with use of the authors’ previously published model was adjusted in a multivariate regression, the effect and level of significance were substantially unaffected (β = 0.08; *p* = 0.02). Findings regarding other cognitive tests and long-term outcome did not reach statistical significance. Nevertheless, the serious limitations of the study are a very small sample size and a presence of the outlier who was excluded from the calculations. Such data should be interpreted carefully, however without any doubts they highlight the need for further studies exploring this issue on a bigger group of patients.

In 2018, a randomized controlled trial conducted by Vedel et al. was published [24]. They recruited 197 patients who were allocated to two different MAP management strategies: one group had their MAP kept around 70–80 mmHg (high-target group) and the second group around 50–60 mmHg (low-target group). Although the primary outcome of the study was the total volume of new ischemic brain lesions, the researchers also examined patients for the occurrence of POCD at median day 7 (range, 3–11 days) and median day 90 (range, 38–180 days) after surgery using ISPOCD test battery. No differences between the two groups were observed, however, as mentioned by the authors themselves, a major limitation of this study is the significant discrepancy of age between high-target and low-target groups (median 69 vs. 65 years). Older age is a known risk factor for the occurrence of POCD.

Available studies on the association between intraoperative hypotension or intraoperative blood pressure drop in cardiac surgery and postoperative cognitive decline (deficit) are summarized in Table 1.

### 3.2. Postoperative Delirium

In another randomized controlled study performed by Siepe et al., 92 patients undergoing elective or urgent on-pump CABG were assigned to either low (60–70 mm Hg) or high (80–90 mm Hg) MAP during CPB [25]. It was found that the postoperative drop in MMSE (Mini-Mental State Examination) score was significantly higher in the low MAP group (3.9 ± 6.5 vs. 1.1 ± 1.9 points; *p* = 0.01). In addition, only subjects in the low MAP group developed postoperative delirium (13% vs. 0%; *p* = 0.017), however the authors defined POD as any score 10 points lower than the preoperative MMSE result, along with positive assessment by a psychologist. Since MMSE is not suited for screening delirium, these results should be interpreted very cautiously [26].

In 2010, Hsiu-Ching et al. performed a study examining the incidence of not only delirium but also subsyndromal delirium (SSD) and cognitive changes among patients undergoing elective CABG [27]. The authors assessed multiple hypothetical risk factors for delirium, such as use and duration of cardiopulmonary bypass, aortic cross-clamp time, intraoperative/postoperative blood transfusion and intraoperative hypotension (defined as MAP < 60 mmHg) time. 38 enrolled patients were screened for POD and SSD daily during the first week after surgery using the CAM (Confusion Assessment Method). Cognitive function was assessed using the MMSE three times: on admission, at discharge and 2–4 weeks post discharge. POD occurred in 18.3%, while SSD in 34.2% of patients. Delirium was more common in participants who received CABG with CPB than off-pump CABG. Most importantly, patients who developed POD/SSD had significantly longer time of intraoperative MAP < 60 mmHg than those without any disfunction ((81.4 (POD) vs 24.6 (SSD) vs. 13.3 min (no delirium)) and received more blood units, what may suggest that hemodynamic instability and thus hypotension plays a role in postoperative brain function. Moreover, individuals with POD and SSD scored lower on the MMSE at hospital discharge than those without delirium. Undoubtedly this study presents an association between hypotension and POD in cardiac surgery patients, however the sample was very small and consisted of mostly men (89.5%).

Another observational study examining the relationship between POD and hypotension included 734 patients after on-pump cardiac surgery [28]. It was nested within a large clinical trial, namely the Dexamethasone for Cardiac Surgery (DECS) trial in which patients were receiving either a single intraoperative dose of dexamethasone or placebo [29]. After the procedure, the patients were screened for delirium using CAM-ICU or CAM scale for four consecutive days. As theoretically insufficient brain perfusion is not only dependent on the depth of hypotension, but its duration as well, IOH was defined as area under the curve (AUC) for a certain MAP threshold and expressed in mmHg*min [8]. In this study, four MAP thresholds were specified: <50 mm Hg, <60 mm Hg, decrease > 30% to baseline MAP and decrease >40% to baseline MAP. Delirious patients had higher median AUCs than non-delirious patients when MAP < 60 mm Hg or MAP < 50 mm Hg thresholds were applied, however the finding did not reach statistical significance. Moreover, after adjusting for confounding and multiple testing, there were no significant associations between IOH based on any of the definitions and delirium. Notwithstanding the above, patients who developed POD required longer vasopressor infusion during the surgery than non-delirious patients (median 119 vs. 46 min, *p* < 0.01), what may indicate that disturbed hemodynamic function and vascular tension play a role in pathophysiology of delirium [30]. Even though, in this study IOH was not associated with the occurrence of POD after cardiac surgery, its certain drawbacks should be taken into account: the authors admit possible omission of patients with hypoactive presentation of POD and uncertainty about including the patients receiving vasopressors in the analysis. As there are multiple theories discussing the abnormal neurotransmission, inflammation and stress response in POD, the administration of dexamethasone raises doubts about its possible influence on the results [28]. However, in a different study nested in DECS trial as well and regarding the same cohort, dexamethasone did not reduce the incidence or duration of delirium [29].

Anderson et al. retrospectively analysed data from 155 patients who underwent lung transplantation between June 2013 and July 2016 in order to find the risk factors and determine the long-term impact of delirium [31]. POD was defined as a presence of the terms “delirium”, “delirious”, “CAM positive” in a patient’s chart or acclaimed treatment with antipsychotics. Overall delirium was diagnosed in 36.8% patients. The duration of time with an intraoperative MAP < 60 mmHg was an independent factor associated with POD. Furthermore, as patients had central venous pressure (CVP) monitored during the surgery, CPP was calculated as CPP = MAP − CVP and its threshold was set for 50 mmHg as a hemodynamic risk factor. There was a statistically significant difference in median duration of CPP < 50 mmHg between delirious and non-delirious patients (109 vs. 39 min; *p* = 0.016), however it was not independently associated with delirium.

In the next study regarding lung transplant recipients Smith et al. calculated CPP (i.e., MAP − CVP) for 63 patients and assessed its relationship with POD [32]. CAM and CAM-ICU tools were applied daily for the first week after the surgery and were discontinued afterwards if the patient had three consecutive negative results. Positive CAM/CAM-ICU result was followed by assessment with Delirium Rating Scale in order to establish POD severity. POD was observed in 37% of patients, mostly in the first three days following the transplant. Full model of delirium risk showed that lower CPP was related with higher incidence of delirium: every 10 mmHg decrease in CPP doubled the odds of POD (OR = 2.08; 95%CI 1.08–4.24; *p* = 0.04). Moreover, there was a correlation between lower CPP and greater severity (b = −0.81; 95%CI −1,47 to −0,15; *p* = 0.017) and longer duration of POD (b = −0.54; 95% CI −1 to −0.08; *p* = 0.02).

Another study assessed the association between BP deviations from optimal MAP and POD in patients undergoing cardiac surgery with CPB [33]. It is particularly interesting, as optimal MAP was established by cerebral blood flow autoregulation monitoring with the use of ultrasound-tagged near-infrared spectroscopy (UT-NIRS) and thus had individual value for each patient. UT-NIRS was performed during the CPB and for the first three hours after admission to the ICU. Delirium was assessed using CAM/CAM-ICU scale on each postoperative day 1–3 by research assistants who completed formal training protocol. If POD was present, its severity was evaluated using Delirium Rating Scale-Revised-98 (DRS-R-98). In total 47 (47.5%) out of 99 analyzed patients developed delirium. It was found that blood pressure fluctuations above and below the optimal MAP during CPB and in the ICU were not significantly associated with incidence of delirium on postoperative days 1 and 3. What is interesting, blood pressure excursions above the optimal MAP correlated with presence and severity of delirium on postoperative day 2. The authors explained this finding with possible vasogenic white matter oedema deriving from transudation of fluid into the pericapillary astrocytes as a result of increased blood pressure, since the similar mechanism is present in nonsurgical patients with acute hypertensive emergencies [34]. Even though the sample size was small and delirium was assessed for only three days after surgery, the above mentioned study shed light on the importance of keeping MAP within appropriate range for cerebral autoregulation.

Similar results were obtained by Hori et al. who applied near-infrared spectroscopy in 491 patients undergoing cardiac surgery with CPB [35]. The authors estimated the limit for CBF autoregulation for each patient by using a monitor calculating a continuous, moving Pearson’s correlation coefficient between MAP and NIRS-derived regional cerebral oxygen saturation, rendering the variable cerebral oximetry index (COx). When CBF autoregulation is efficient, COx is near 0 or has a negative value, whereas in case of blood pressure above or below the autoregulation limits, COx increases towards 1. The limit of autoregulation was defined as that MAP at which COx increased from <0.3 to >0.3, as such a cut-off point has been described as the one with the best sensitivity and specificity for identifying the borders of autoregulation [36]. Delirium was recognized if any of the following observations made by clinical staff was present: delirium, confusion, agitation, or change in mental status. There was a significant difference in POD occurrence in patients whose MAP exceeded the upper limit of autoregulation than in patients whose MAP did not surpass the upper limit (12.9 vs. 3.2%, *p* < 0.001). Moreover it was found that the sum of the magnitude and duration of MAP above the upper threshold of cerebral autoregulation during CPB had a statistically significant association with the risk of delirium (OR = 1.09; 95%CI = 1.03–1.15; *p* < 0.001). No similar results were obtained in case of MAP below the autoregulation threshold. Despite its certain limitations, such as imprecise definition of delirium, this study demonstrates that too high MAP can result in adverse neuropsychological events and emphasizes the importance of individualized approach in establishing optimal target MAP for the patient.

Krzych et al. processed data from 5781 patients operated in high-volume cardiac surgery centre with the aim to investigate the incidence and risk factors of postoperative delirium [14]. POD was diagnosed using DSM-IV criteria by the attending cardiac surgeon or intensive care specialist and verified by a consulting psychiatrist afterwards. Among 100 perioperative patient-specific and treatment variables analysed by the authors, some independent determinants in multivariate analysis are worth emphasising in the context of reduced cerebral blood flow. In addition, protective effect of hypertension was found in contrary to most of the recent studies, however the authors explained this phenomenon with possible influence of appropriate BP control before the surgery, protective effect of pharmacological agents (such as angiotensin converting enzyme inhibitors) and decreased variations of blood pressure during CPB.

Vedel et al. recruited 197 patients requiring CBP for the randomized controlled trial aimed to explore the occurrence of brain ischemic lesions after surgery [24]. The patients were divided into two MAP management groups: one, the so-called high-target group, had their MAP kept around 70–80 mmHg, whereas the second group, low-target, had their MAP kept around 50–60 mmHg. The researchers screened patients for delirium and hallucinations, however no delirium screening method was provided by the authors, as they acquired this data from medical records. No significant difference in terms of delirium frequency among the two groups was observed (7.1% in low-target group vs. 10.5% in high-target group, *p* = 0.45). Again, as mentioned in the POCD section of this systematic review, the high-target group was significantly older than the low-target group (median 69 vs. 65 years). 

Finally, in 2019 Brown et al. shared the results of their randomized controlled trial aimed to determine whether targeting MAP during CPB by monitoring cerebral autoregulation can reduce the incidence of delirium compared with usual care [37]. In the intervention group (105 participants), the patient’s MAP was maintained above the lower limit of cerebral autoregulation (established with use of transcranial doppler monitoring of the middle cerebral arteries after initiating the surgery). In the control group (94 participants), conventional blood pressure management strategy was applied. For the first four days after the procedure the patients were screened for delirium with use of CAM/CAM-ICU and any potential diagnosis was confirmed by consensus panel using DSM-V criteria. POD was recognized in 38% patients from the intervention group versus 53% from control group (*p*  = 0 .04). Moreover, the odds for the occurrence of delirium were reduced by 45% in patients allocated to the intervention group (odds ratio, 0.55; 95% CI, 0.31–0.97; *p* =  0.04).

Available studies on the association between intraoperative hypotension (or intraoperative blood pressure drop-in cardiac surgery and postoperative cognitive delirium are summarized in Table 2.

## 4. Limitations

Although the pathophysiological link between hypotension, hypoperfusion and neurological complications is clear, it is difficult to demonstrate it in clinical trials, especially in terms of cognitive function. It requires to consider a number of pre- and perioperative factors, often immeasurable and individually variable. The available scientific evidence is limited by the lack of definitions and uniform diagnostic criteria for POCDs.

The studies differed in terms of the selection of the tests used, their number and the criteria for determining the deterioration of the cognitive domain. Gold et al. [17] defined the deterioration as a worse result in at least 3 tests performed, while Siepe et al. [25] when the MMSE score was <10 points. From a clinical point of view, this approach is not wrong. Delirium is the most common postoperative neurological complication, so until foreclosure, any cognitive deterioration, regardless of how it is diagnosed, should be treated as delirium and verified by DSM criteria (gold standard). In the Anderson’s et al. study [31], POD was determined retrospectively, based on a history of use of antipsychotic drugs in the medical records. Only some POD patients were diagnosed with the CAM algorithm. Hori et al. [33] recognized POD based on the opinion of a psychiatrist. The abovementioned heterogeneity in available studies makes it difficult for multicenter data comparisons. Furthermore, it cannot be excluded that the patient is deteriorating in a certain cognitive area and improving in another, what ultimately enhances the quality of life [38]. Additionally, a successful cardiac surgery procedure may result in discontinuation of cognitive-sensitive drugs, which makes it problematic to assess the relationship between IOH and POCD. The discussion on the optimal time for carrying out the control tests in the postoperative period remains open. Gold and Charlson et al. [17,19] evaluated patients in the first week and after 6 months, Siepe et al. [25] performed MMSE test 48 h after surgery, and Larsen et al. [20] performed a 3-year follow-up. On the one hand, repeating the same tests in a short time intervals creates a risk that the patient is under the influence of perioperative drugs or achieves a better result due to the effect of “learning” the test. Moreover, an assessment too distant over time may show a deterioration unrelated to perioperative complications, but would reflect the progression of concomitant diseases or the development of senile dementia [39]. On average, it has been shown that cognitive recovery occurs in the 3rd month after surgery [38]. Moreover, it cannot be excluded that only patients in better condition survive, until a distant psychological re-evaluation, which will distort the results in the study population.

The heterogeneity of the research was also due to how IOH was defined. The lack of uniform criteria was a reason for discrepancy among researchers. The definitions referred to a specific absolute value or a percentage deviation from the accepted baseline value (MAP or systolic pressure, SBP). However, the baseline MAP or SBP should be determined on an outpatient basis at the planning stage of the procedure. The use of pre-induction measurement as a baseline for intraoperative blood pressure management is questionable [40]. Only Wesselink et al. applied complex IOH recognition criteria, but still based on arbitrarily established cut-off points [28]. Hori et al. made progress in intraoperative hemodynamic management [33], in which they used NIRS to continuously evaluate cerebral autoregulation during and after surgery, however this practice is not available everywhere and requires further research. Regardless of how the authors defined IOH, different strategies were used to achieve the hemodynamic goals, i.e., the use of fluid therapy, vasoactive drugs (administered in bolus or continuous infusions). The specificity of cardiac surgery, where CPB is often implemented, may require a different approach to the hemodynamic management. Meng et al. [9] recommend MAP to be maintained within the wide limits of 70–100 mmHg during CPB, while for the remaining period of the procedure, the MAP should be kept within 90–110% of the baseline values. However, this latter recommendation should be adopted with caution as it was based on data from non-cardiac surgery [41]. In addition, maintaining MAP within 90–110% of the baseline was implemented only for the intraoperative period and 4 h after its completion. Further hemodynamic management after this period undoubtedly has a significant impact on POCD, especially that cardiac surgical patients are a population particularly vulnerable to postoperative hemodynamic instability. Anderson et al. rightly noted that brain perfusion, in addition to MAP, is affected by venous return [31]. CVP values may be subject to significant changes during the perioperative period due to body position, fluid therapy and even operator maneuvers. The calculated CPP value considers, albeit only mathematically, the relationship between MAP and CVP, which is why it is a more optimal parameter for assessing cerebral perfusion [30]. However, one ought to remember that CVP cannot be used as a surrogate for ICP. This approach has several limitations, i.e., in patient with right ventricle insufficiency or chronic obstructive pulmonary disease. Krzych et al. also pointed to a significant link between stenosis in the arteries supplying the brain and the development of POD, suggesting that future research on IOH should routinely take this clinical aspect into account [14]. Finally, we failed to explore the causes that lead to hypotension in this setting. Unfortunately, the cited papers were too diverse in their methodology to even try to investigate the reasons of IOH in a systematic manner. It may arise from reperfusion injury after cardioplegin-induced cardiac arrest, vasoplegic shock developing after discontinuation of CBP, low cardiac output due to impairment of LV/RV function, on-going myocardial ischemia or acute-on-chronic pulmonary hypertension, and many others. We also did not attempt to identify patients at risk of IOH and how to prevent it in an evidence-based approach. The plethora of predisposing and precipitating factors of delirium has been identified so far in prospective studies. However, the majority of projects included in our review was retrospective in nature, in which the endpoint (POD, POCD) was assessed in relation to the assumed definition of IOH, which is a limitation of concluding with regard to other risk factors. In randomized trials, vasoactive drugs were used to maintain previously established haemodynamic goals.

Finally, currently, there are no ongoing studies registered in ClinicalTrials.gov database designed to evaluate the effects of IOH on POD and POCD.

## 5. Conclusions

Available scientific data speak against intraoperative management of arterial pressure on the basis of arbitrarily accepted cut-off points. Therapy should be tailored to the individual needs of the patient and based on reliable, advanced hemodynamic monitoring. Only by ensuring such standardization of the procedure will it be possible to compare the results obtained between the centers. The assessment of the relationship between intraoperative hypotension and cognitive impairment is difficult due to the multitude of interfering factors and requires further research.

## Figures and Tables

**Table 1:** Summary of studies on the association between intraoperative hypotension (IOH) or intraoperative blood pressure drop in cardiac surgery and postoperative cognitive decline (deficit) (POCD).

Author	Design of the Study	Study Population	IOH Definition	POCD Screening Method	Effect
Gold et. al. [17]	Randomized controlled	248 patients undergoing elective CABG with CPB	Two BP management strategies:MAP between 80–100 mmHg or 50–60 mmHg	Battery of 11 neuropsychological tests applied preoperatively, 7 days and 6 months post procedure	No effect at 6 months follow-up
Charlson et. al. [19]	Randomized controlled	412 patients undergoing elective CABG with CPB	Two BP management strategies: MAP target determined by patient’s usual pre-bypass blood pressure or “high” MAP maintained around 80 mmHg	Battery of 11 neuropsychological tests applied preoperatively, on the 1–2 and 5–6 postoperative days and at 6 months follow-up	No effect
Larsen et. al. [20]	Randomized controlled	113 patients undergoing cardiac surgery with CPB	Two BP management strategies: high target MAP = 70–80 mmHg or low target MAP = 40–50 mmHg	MMSE, ISPOCD test battery and three additional questionnaires applied before the surgery and at 3 years follow-up	No effect
Newman et.al. [21]	Cohort	237 patients undergoing elective cardiac procedure with CBP	MAP < 50 mm Hg	Neuropsychologic test battery on the day prior to the surgery and the day prior to the hospital discharge	Low intraoperative MAP contributes neuropsychologic dysfunction in elderly
Gottesman et. al. [23]	Cohort	15 patients with high risk for postoperative stroke undergoing elective on-pump CABG	Not defined	The neurocognitive examination consisted of MMSE, Trail Making Test A and B and the modified Rankin Scale applied 3–5 days after surgery and after 1 month	Each additional point decrease in baseline MAP during the surgery led to a 0.09-point greater decline in early postoperative MMSE score
Vedel et al. [24]	Randomized controlled	197 patients undergoing cardiac surgery with CBP	Two BP management strategies: a strategy of high target for MAP (70–80 mmHg) and strategy of low target for MAP (40–50 mmHg)	ISPOCD test battery used at median day 7 and median day 90 after the procedure	No significant difference was observed at either 7 and 90 day after procedure

**Table 2:** Summary of studies on the association between intraoperative hypotension (IOH) or intraoperative blood pressure drop in cardiac surgery and postoperative cognitive delirium (POD).

Author	Design of the Study	Study Population	IOH Definition	POD Screening Method	Effect
Siepe et al. [25]	Randomized controlled	92 patients undergoing elective or non-elective on-pump CABG	Intervention group with MAP maintained between 60–70 mmHg	Postoperative (48 h after procedure) MMSE score 10 points lower than the preoperative assessment	Only patients with MAP kept between 60–70 mmHg exhibited POD
Hsiu-Ching et al. [27]	Prospective observational	38 patients undergoing elective on-pump or off-pump CABG	MAP < 60 mmHg	Daily CAM assessment during the first week after surgery	Patients who developed POD experienced longer duration of intraoperative MAP < 60 mmHg
Wesselink et al. [28]	Observational study nested in another clinical trial	734 patients undergoing on-pump cardiac surgery	Four definitions were explored: MAP < 50 mmHg, MAP < 60 mmHg, 30% and 40% decrease of baseline MAP values	Daily CAM assessments for four consecutive days after procedure	No effect regarding the hypotension thresholds
Anderson et al. [31]	Retrospective observational	155 patients undergoing lung transplantation	MAP < 60 mmHg	Presence of terms: “delirious”, “delirium”, “CAM positive” or acclaimed antipsychotic treatment in medical records of hospitalization	The duration of time with an intraoperative MAP < 60 mmHg was an independent risk factor for development of POD
Smith et al. [32]	Prospective observational	63 patients undergoing lung transplantation	Not defined	Daily CAM or CAM-ICU assessments for the first 7 days after surgery	Lower CPP was related with higher incidence of delirium: every 10 mmHg decrease in CPP doubled the odds of POD
Hori et al. [33]	Prospective observational	99 patients undergoing on-pump cardiac surgery	Not defined	CAM or CAM-ICU assessments during the first 3 days after surgery	Blood pressure excursions above the optimal MAP correlated with presence and severity of delirium on postoperative day 2
Hori et al. [35]	Prospective observational	491 patients undergoing on-pump cardiac surgery	Not defined	Observations made by a clinical staff	Higher POD occurrence was observed in patients whose MAP exceeded upper limit of cerebrovascular autoregulation
Krzych et al. [14].	Prospective observational	5781 patients undergoing on-pump and off-pump cardiac surgery	Not defined	DSM-IV evaluations done by an attending physician and a psychiatrist	No effect
Vedel et al. [24]	Randomized controlled	197 patients undergoing cardiac surgery with CPB	Two BP management strategies: a strategy of high target for MAP (70–80 mmHg) and strategy of low target for MAP (40–50 mmHg)	No screening tests provided; the data was acquired via medical records	No effect
Brown et al. [37]	Randomized controlled	199 patients undergoing cardiac surgery with CPB	Two BP management strategies: standard care vs. MAP maintained above individual limit of cerebral autoregulation	CAM or CAM-ICU for the first 4 postoperative days followed by panel discussion considering DSM_V criteria	POD occurred in 38% patients from the intervention group versus 53% in control. The odds for POD were reduced by 45% in patients in the intervention group.

## Data Availability

Not applicable.

## Appendix A

### Search Strategy

#1. PubMed search on 06/01/2021 using ‘MeSH* terms’ & ‘best match’ strategy:

((“Neurocognitive Disorders”[Mesh]) AND “Hypotension”[Mesh]) AND “Surgical Procedures, Operative”[Mesh]) (*n =* 47) OR ((“Intraoperative Period”[Mesh]) AND “Hypotension”[Mesh]) AND “Treatment Outcome”[Mesh]) (*n =* 17) OR ((“Perioperative Period”[Mesh]) AND “Hypotension”[Mesh]) AND “Outcome Assessment, Health Care”[Mesh]) (*n =* 40) OR ((“Treatment Outcome”[Mesh]) AND “Intraoperative Period”[Mesh]) AND “Hypotension”[Mesh]) (*n =* 17) OR ((“Intraoperative Period”[Mesh]) AND “Blood Pressure”[Mesh]) AND “Neurobehavioral Manifestations”[Mesh] (*n =* 7)

* MeSH—Medical Subject Headings.

#2. PubMed search on 06/01/2020 using ‘key words’ & ‘best match’ strategy:

((intraoperative hypotension) AND (postoperative period)) AND (delirium) (*n =* 16) OR (intraoperative hypotension) AND (cognitive defect) (*n =* 3) OR ((perioperative) AND (hypotension)) AND (cognitive function) (*n =* 21) OR ((intraoperative) AND (hypotension)) AND (cognitive impairment) (*n =* 25) OR (intraoperative blood pressure) AND (delirium) (*n =* 85) OR (intraoperative hypotension) AND (outcome) (*n =* 1568) OR ((surgery) AND (blood pressure)) AND (cognition) (*n =* 994) OR (postoperative cognitive dysfunction) AND (blood pressure) (*n =* 149) OR (hypotension) AND (postoperative cognitive dysfunction) (*n =* 85)

#3. Embase search on 06/01/2020 using ‘all fields’ algorithm:

(‘perioperative period’/exp OR ‘perioperative period’) AND (‘cognition’/exp OR cognition) AND (‘blood pressure’/exp OR ‘blood pressure’) (*n =* 207) OR ‘cognitive defect’ AND ‘intraoperative hypotension’ (*n =* 9) OR ‘hypotension’ AND ‘intraoperative period’ AND ‘cognition’ (*n =* 243) OR ‘hypotension’ AND ‘intraoperative period’ AND ‘disorders of higher cerebral function’ (*n =* 275) OR ‘treatment outcome’ AND ‘intraoperative hypotension’ (*n =* 173) OR ‘postoperative cognitive dysfunction’ AND ‘hypotension’ (*n =* 96)

#4 Cochrane Library search on 06/01/2020:

((intraoperative hypotension) AND (postoperative period)) AND (delirium) (*n =* 19) OR (intraoperative hypotension) AND (cognitive defect) (*n =* 3) OR ((perioperative) AND (hypotension)) AND (cognitive function) (*n =* 16) OR ((intraoperative) AND (hypotension)) AND (cognitive impairment) (*n =* 11) OR (intraoperative blood pressure) AND (delirium) (*n =* 90) OR (intraoperative hypotension) AND (outcome) (*n =* 679) OR ((surgery) AND (blood pressure)) AND (cognition) (*n =* 298) OR (postoperative cognitive dysfunction) AND (blood pressure) (*n =* 81) OR (hypotension) AND (postoperative cognitive dysfunction) (*n =* 30)

#5 OpenGrey search on 06/01/2020:

((intraoperative hypotension) AND (postoperative period)) AND (delirium) (*n =* 0) OR (intraoperative hypotension) AND (cognitive defect) (*n =* 0) OR ((perioperative) AND (hypotension)) AND (cognitive function) (*n =* 0) OR ((intraoperative) AND (hypotension)) AND (cognitive impairment) (*n =* 0) OR (intraoperative blood pressure) AND (delirium) (*n =* 0) OR (intraoperative hypotension) AND (outcome) (*n =* 0) OR ((surgery) AND (blood pressure)) AND (cognition) (*n =* 0) OR (postoperative cognitive dysfunction) AND (blood pressure) (*n =* 0) OR (hypotension) AND (postoperative cognitive dysfunction) (*n =* 0)

#6 ClinicalTrials.gov search on 06/01/2020:

Delirium & cardiac surgery: recruiting (*n =* 28), completed (*n =* 43), withdrawn (*n =* 2), unknown (*n =* 12), terminated (*n =* 6), active & non-recruiting (*n =* 9), not yet recruiting (*n =* 5)

POCD—Postoperative Cognitive Dysfunction & cardiac surgery: recruiting (*n =* 13), completed (*n =* 12), unknown (*n =* 9), terminated (*n =* 3), active & non-recruiting (*n =* 3), not yet recruiting (*n =* 2)
